# Factors associated with Iraqi dental students’ intention to perform dental implant therapy after graduation: a cross-sectional study

**DOI:** 10.1186/s12903-026-09014-x

**Published:** 2026-07-21

**Authors:** Hani Radhi, Qasiar Khaleel Oraibi, Ahmad Fliah Hassan, Faaiz Yaqub Alhamdani

**Affiliations:** 1 Oral and Maxillofacial Surgery, Department of Oral Surgery and Periodontics, Mustansiriyah University, Baghdad, Iraq; 2 Oral and Maxillofacial Surgery, Department of Oral and Maxillofacial Surgery, College of Dentistry, Al-Esraa University, Baghdad, Iraq; 3 Oral Medicine and Pathology, Oral Medicine Department, College of Dentistry, Mustansiriyah University, Baghdad, Iraq; 4 Oral and Maxillofacial Surgery, Oral & Maxillofacial Surgery and Oral Diagnostics Department, College of Dentistry, Ibn Sina University of Medical and Pharmaceutical Sciences, Baghdad, Iraq

**Keywords:** Dental implants, Education, Dental, Students, Dental, Cross-sectional studies, Logistic models, Treatment planning, Undergraduate dental education, Implantology education, Professional intention

## Abstract

**Objectives:**

This study aimed to identify the demographic, educational, and perceptual factors associated with undergraduate dental students’ intentions to perform dental implant therapy after graduation.

**Methods:**

A cross-sectional analytical study was conducted during the 2024–2025 academic year among fourth- and fifth-year dental students (*n* = 304). A structured online questionnaire collected data on demographic characteristics, sources of implant-related information, perceived importance of dental implants, preferred management of severely compromised teeth, views on the delivery of the implantology curriculum (stand-alone versus integrated), and self-reported familiarity with implant systems. Associations were examined using chi-square tests and multivariable binary logistic regression (α = 0.05).

**Results:**

Overall, 65.8% of the respondents (200/304) indicated an intention to perform implant therapy after graduation. In the adjusted logistic regression model, intention was higher among students who preferred extraction followed by implant placement than among those who preferred root canal treatment with build-up (AOR = 2.26; 95% CI 1.24–4.12; *p* = 0.008). Lower odds of intention were observed among female students (AOR = 0.49; 95% CI 0.25–0.94; *p* = 0.033), students who rated implants as “very important” (AOR = 0.27; 95% CI 0.12–0.60; *p* = 0.001) or “important” (AOR = 0.42; 95% CI 0.18–1.00; *p* = 0.049) compared with “essential,” and students who did not support implantology as a separate subject (AOR = 0.51; 95% CI 0.27–0.96; *p* = 0.038).

**Conclusions:**

Future intention to perform dental implant therapy was associated mainly with treatment preference, perceived importance of implant therapy, and views on implantology teaching as a separate subject, rather than academic stage or informal learning sources. These findings suggest that clinically meaningful undergraduate implant education may be more closely associated with professional intention than exposure alone.

**Supplementary Information:**

The online version contains supplementary material available at https://doi.org/10.1186/s12903-026-09014-x.

## Introduction

Dental implant treatment has become an essential component of modern oral rehabilitation. The usage rate has increased substantially over the past decades, and predictions indicate that this trend will continue [1]. Alongside these changes, advances in biomaterials, surface technologies, and digital planning systems have increased the predictability and indications for treatment [2]. In Iraq, implant therapy has been clinically assessed, indicating its relevance in the local restorative landscape [3]. As implant treatment is steadily assimilated into general dental practice, the preparedness of undergraduate dental graduates is crucial.

Formal recommendations for implant dentistry in preclinical curricula have existed for over three decades, based on the view that implant dentistry is essential for comprehensive oral rehabilitation and should therefore be introduced at the undergraduate level [4, 5]. Later European consensus workshops further specified that undergraduate students should achieve competence in diagnosis, treatment planning, and restoration of straightforward implant cases, while complex surgical procedures should remain within postgraduate training [6–8]. Despite these recommendations, substantial variability in implant education persists across European institutions [9, 10]. International studies have generally reported positive student attitudes toward implant dentistry, although deficiencies in hands-on training, confidence, and perceived preparedness remain common [11–18]. Digital technology and artificial intelligence are also increasingly influencing implant diagnosis, treatment planning, and education, and students appear broadly receptive to their integration into training [19–22]. Recent evidence from Malaysian clinical-year dental students also supports the relevance of artificial-intelligence-related perceptions in dental education [23]. In undergraduate implant education, a distinction should be made between simple exposure and competence-oriented learning. Exposure refers to encountering implant-related information through lectures, videos, workshops, social media, or informal observation. In contrast, clinically meaningful or competence-oriented implant education refers to structured learning that helps students apply implant-related knowledge to diagnosis, case selection, treatment planning, restoration of straightforward cases, recognition of surgical complexity, risk assessment, maintenance, and appropriate referral. This distinction is important because professional intention may be associated not only with whether students have heard about implants, but also with whether they perceive implant dentistry as a clinically organised and teachable component of undergraduate care.

In the Iraqi undergraduate dental context, implant-related teaching is generally encountered within broader clinical disciplines rather than through a uniformly standardised national stand-alone implantology curriculum. Therefore, students’ perceptions of whether implant dentistry should be taught as a separate subject or integrated with other clinical subjects may reflect broader uncertainty about how implant competencies should be positioned within undergraduate training. This local context provides the rationale for examining curriculum-related perceptions alongside demographic and treatment-planning factors.

In this context, the present study aimed to identify the demographic, educational, and perceptual factors associated with dental students’ intention to perform dental implant therapy after graduation. The null hypothesis was that educational exposure, curriculum structure, and treatment philosophy would not be associated with the future intention to perform dental implant therapy.

## Materials and methods

### Ethical approval

This cross-sectional study was approved by the Institutional Review Board (IRB) of Ibn Sina University of Medical and Pharmaceutical Sciences, Baghdad, Iraq (approval no. ISU.18.1.24; 29 October 2024) and was conducted in accordance with the ethical principles of the Declaration of Helsinki [24]. The participants were informed about the study’s purpose, voluntary nature, and confidentiality before participation. Electronic informed consent was obtained prior to questionnaire completion. This cross-sectional study was prepared in accordance with the STROBE recommendations; the completed checklist is provided in [Supplementary File 1].

### Study design and participants

A cross-sectional analytical study was conducted among fourth- and fifth-year undergraduate dental students during the 2024–2025 academic year. Eligible participants were students enrolled in the fourth or fifth year of the undergraduate dental program who provided electronic informed consent and submitted a complete, non-duplicate, interpretable questionnaire. These academic stages were selected because students at this level are more likely to have been exposed to clinical decision-making and implant-related teaching than earlier-stage students.

No formal a priori sample size calculation was performed prior to data collection. The questionnaire was distributed by three researchers via predefined electronic student channels, and the responses were collected throughout the study period. A total of 542 submissions were received; 304 met the eligibility and data quality criteria and were included in the final complete-case analysis. The participant characteristics are presented in Table 1.

**Table 1:** Participant characteristics (*N* = 304)

Variable	Category	*n*	%
Gender	Male	78	25.7
	Female	226	74.3
Academic Stage	Fourth	66	21.7
	Fifth	238	78.3
Information Source	Videos	108	35.5
	Books/articles	68	22.4
	Social media	74	24.3
	Workshops	40	13.2
	None	14	4.6
Perceived Importance	Essential	62	20.4
	Very Important	146	48.0
	Important	90	29.6
	Not Important	6	2.0
Preferred Treatment	Root canal treatment (RCT) with build-up	176	57.9
	Extraction → Implant	116	38.2
	Extraction → Bridge	6	2.0
	Extraction → Denture	6	2.0
Separate Subject	Yes	232	76.3
	No	72	23.7
Integrated Teaching	Yes	206	67.8
	No	98	32.2
Implant Systems Known	One	48	15.8
	More than one	56	18.4
	None	200	65.8
Future Implant Plan	Yes	200	65.8
	No	104	34.2

Data are presented as frequencies (*n*) and percentages (%) of the total sample (*N* = 304). “Separate Subject” indicates a preference for implant dentistry as an independent course, whereas “Integrated Teaching” indicates its integration with other clinical disciplines. “Implant Systems Known” reflects the self-reported familiarity with implant systems. “Future Implant Plan” represents the intention to perform implant therapy after graduation

### Instrument development and data collection

A structured electronic questionnaire was developed using Google Forms and distributed via institutional emails and social media platforms. The questionnaire was developed specifically for this study based on the study objectives and themes identified in previous implant education literature, including students’ information sources, perceived importance of implant therapy, treatment-planning preferences, views on curriculum structure, self-reported familiarity with implant systems, and intention to perform implant therapy after graduation. It was not adapted from a single previously validated scale. Therefore, the instrument was treated as a structured descriptive survey rather than a psychometric scale, and this limitation is acknowledged in the Limitations section. Although respondent names were collected and used during data screening to identify duplicate submissions, recruitment was conducted via open electronic student channels rather than a closed, individualised invitation list. The survey link was also distributed through multiple open platforms, including student groups and social media channels, and was not restricted to a fixed institutional roster from selected colleges. Therefore, the number of students who received, viewed, or had access to the survey invitation could not be verified, and the eligible source population could not be reliably defined. For this reason, neither a conventional response rate nor an approximate institutional response proportion was calculated. Reporting a denominator based only on selected institutions would risk misrepresenting the survey reach because students from other dental colleges may also have accessed the questionnaire.

All variables were self-reported. Gender was recorded as male or female, and academic stage as fourth or fifth year. Educational-exposure variables included the stage at which implant topics were studied (fourth and fifth stages vs. fifth stage only), the preferred stage to begin implant education (fourth vs. fifth stage), and the main source of implant-related information (videos, books/articles, social media, workshops, or none). Perceptual variables included perceived importance of dental implants (essential, very important, important, or not important) and preferred management of a severely compromised tooth (root canal treatment with build-up, extraction followed by implant, extraction followed by bridge, or extraction followed by denture). Curriculum-structure variables included preference for implant dentistry as a separate subject (yes/no) and preference for integrated teaching with other subjects (yes/no). Implant knowledge was assessed by self-reported familiarity with implant systems (one, more than one, or none). The primary outcome variable was the intention to perform dental implant therapy after graduation (yes/no). The questionnaire is provided in [Supplementary File 2].

Because recruitment used open electronic distribution channels, the sample may have been subject to self-selection bias. To improve data quality, all submitted forms were screened independently by three researchers after the collection period ended. Duplicate submissions were checked using the respondent’s name, academic stage, and institution. Submissions were excluded if they were outside the target academic stages, duplicated, substantially incomplete, lacked interpretable responses for the primary outcome or key covariates, or contained response patterns that could not be interpreted reliably on review. Of the 542 submitted forms, 238 were excluded during screening and 304 were retained for the final complete-case analysis. Because reason-specific exclusion counts were not prospectively retained as separate analytic categories, the exact numbers for each exclusion reason could not be reconstructed reliably. However, the excluded submissions were related to eligibility and data-quality screening, including responses outside the target academic stages, duplicate submissions, substantial incompleteness, missing or uninterpretable responses for the primary outcome or key covariates, and response patterns that could not be interpreted reliably. Therefore, the high proportion of exclusions is acknowledged as a potential source of selection bias. As participants were recruited through open electronic channels rather than a probability-based sampling frame, no weighting procedure or complex sampling adjustment was applied; all analyses were conducted on the final eligible complete-case dataset as an unweighted convenience sample.

### Statistical analysis

Statistical analysis was performed using jamovi (version 2.7.26; The jamovi project, Sydney, Australia) [25, 26]. Descriptive statistics were calculated as frequencies and percentages. Incomplete questionnaires were excluded during screening; therefore, only complete eligible responses were included in the final analysis. Missing data were not tabulated separately by variable among the excluded questionnaires. Because no formal a priori sample size calculation was performed, the adequacy of the final sample was considered pragmatically in relation to the planned analyses. The final analytic sample included 304 students, of whom 200 reported an intention to perform dental implant therapy after graduation and 104 did not. The multivariable logistic regression model was estimated on 288 students after exclusion of sparse categories. The number of outcome events was considered acceptable for the number of predictors retained in the final model; however, the analysis should still be interpreted cautiously because the sample was based on convenience recruitment and was not powered a priori for all subgroup comparisons.

Associations between categorical variables and intention to perform implant therapy after graduation were assessed using the chi-square test of independence, and effect sizes were calculated using Cramér’s V. For 2 × 2 comparisons, continuity-corrected chi-square values were reported. When expected cell counts were small, low-frequency categories were considered in the interpretation and, where relevant, excluded from multivariable modelling to avoid unstable estimates.

A multivariable binary logistic regression model was constructed to identify independent predictors of intention to perform implant therapy after graduation. Variables entered into the multivariable model were selected based on their relevance to the study question and their observed association in the bivariate analysis. The final model included sex, perceived importance of dental implants, preferred treatment approach for a severely compromised tooth, preference for implant dentistry as a separate subject, and self-reported familiarity with implant systems. Categorical predictors were entered using reference-category coding. Male sex, essential importance, root canal treatment with build-up, support for implant dentistry as a separate subject, and familiarity with one implant system were used as reference categories. Because of very small frequencies, the categories extraction followed by bridge, extraction followed by denture, and not important were excluded from the multivariable model to avoid unstable estimates. A sensitivity analysis additionally recoded implant-system familiarity as “any familiarity” versus “none”. No subgroup or interaction analyses were performed. Adjusted odds ratios (AORs) with 95% confidence intervals (CIs) were reported, and statistical significance was set at *p* < 0.05.

## Results

### Participant characteristics

A total of 542 questionnaire submissions were received. After screening for eligibility, duplicate submissions, completeness, and interpretability, 304 responses were retained for the final complete-case analysis, and 238 were excluded. Among the 304 included students, 200 (65.8%) reported intending to perform dental implant therapy after graduation. The sample was predominantly female and fifth-year students, which should be considered when interpreting subgroup comparisons.

### Bivariate associations

Bivariate associations between the explanatory variables and intention to perform dental implant therapy were evaluated using the chi-square test (Table 2).

**Table 2:** Bivariate associations with implant practice intention

Predictor	χ²	*p*-value	Cramér’s V
Treatment Preference	15.21	0.0016	0.224
Perceived Importance	10.07	0.0179	0.182
Integration with Other Subjects	6.66	0.0099	0.148
Separate Subject	6.36	0.0117	0.145
Implant Systems Known	8.71	0.0129	0.169
Gender	5.13	0.0235	0.130
Academic Stage	0.37	0.5421	0.035
Study Topic Stage	1.06	0.3040	0.059
Preferred Start Stage	1.96	0.1620	0.080
Information Source	5.24	0.2639	0.131

Chi-square tests of independence were used to examine the associations between categorical variables and the intention to perform dental implant therapy. Effect size was assessed using Cramér’s V. A two-sided *p*-value < 0.05 was considered statistically significant

### Multivariable logistic regression

The multivariable logistic regression model was statistically significant (likelihood ratio test, *p* < 0.001) and was estimated for 288 students after exclusion of categories with very small frequencies (Table 3).

**Table 3:** Multivariable logistic regression predicting intention to perform implant therapy after graduation

Predictor	AOR	95% CI	*p*-value
Female (vs. Male [Ref])	0.49	0.25–0.94	0.033
Very Important (vs. Essential [Ref])	0.27	0.12–0.60	0.001
Important (vs. Essential [Ref])	0.42	0.18–1.00	0.049
Extraction → Implant (vs. RCT + build-up [Ref])	2.26	1.24–4.12	0.008
Not Separate Subject / No (vs. Yes [Ref])	0.51	0.27–0.96	0.038
> 1 Implant System (vs. One [Ref])	0.33	0.12–0.91	0.032
None (vs. One [Ref])	0.36	0.15–0.84	0.019

A multivariable binary logistic regression model was constructed to identify the independent predictors of the intention to perform implant therapy after graduation. In the adjusted model, preference for extraction followed by implant rather than root canal treatment with build-up was associated with higher odds of intending to perform implant therapy after graduation (AOR = 2.26; 95% CI 1.24–4.12; *p* = 0.008). Lower odds were observed among female students compared with male students (AOR = 0.49; 95% CI 0.25–0.94; *p* = 0.033), among students rating implant therapy as “very important” (AOR = 0.27; 95% CI 0.12–0.60; *p* = 0.001) or “important” (AOR = 0.42; 95% CI 0.18–1.00; *p* = 0.049) compared with “essential,” and among students who did not support implantology as a separate subject (AOR = 0.51; 95% CI 0.27–0.96; *p* = 0.038). Students who reported familiarity with more than one implant system (AOR = 0.33; 95% CI, 0.12–0.91; *p* = 0.032) or no implant system (AOR = 0.36; 95% CI, 0.15–0.84; *p* = 0.019) also had lower odds in the primary model. However, in a sensitivity analysis in which implant-system familiarity was recoded as any familiarity versus none, implant familiarity was not associated with intention to perform implant therapy after graduation (AOR = 1.25; 95% CI, 0.65–2.39; *p* = 0.502), and this finding was interpreted with caution

## Discussion

**Fig. 1:**
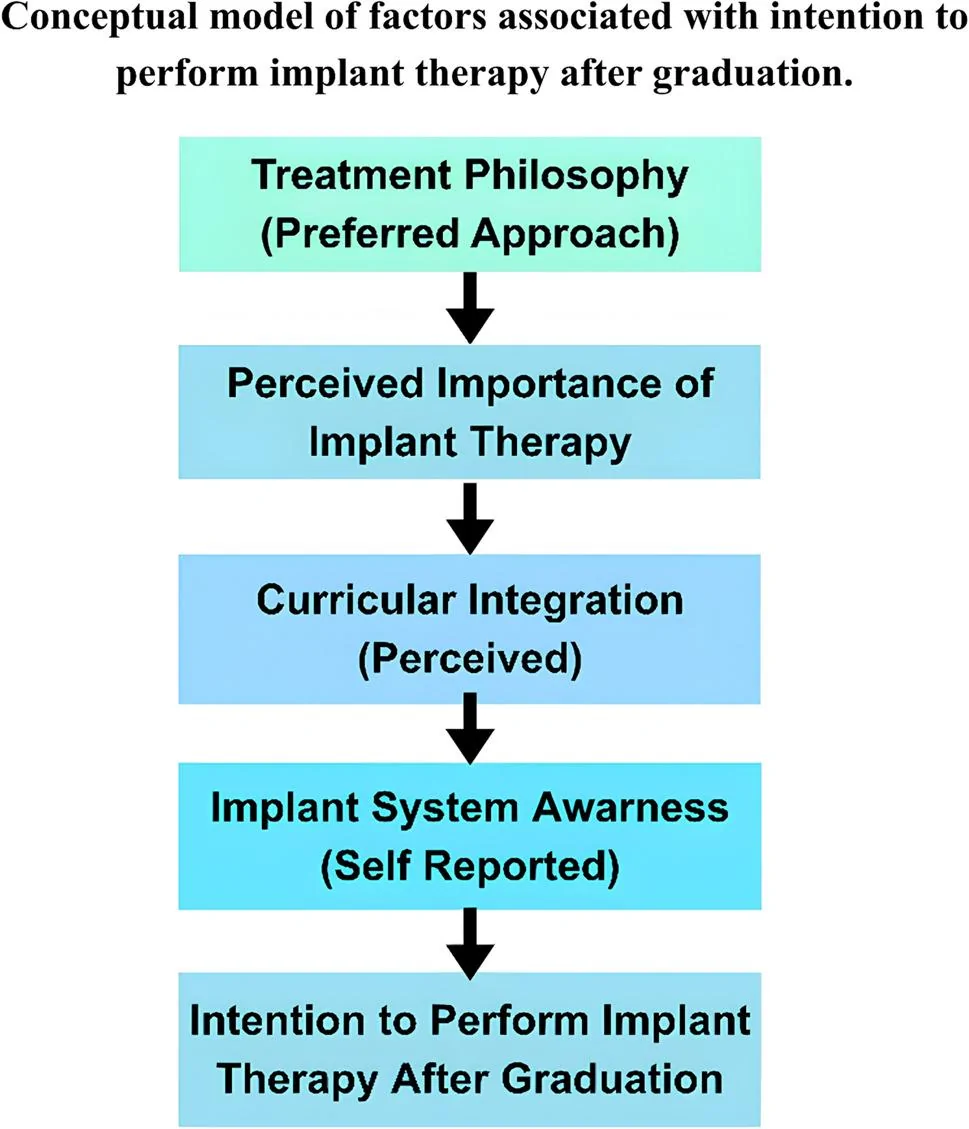
Conceptual model of factors associated with the intention to perform implant therapy after graduation. The figure summarises the factors associated with intention in the multivariate analysis and should be interpreted as an association-based conceptual summary rather than a causal pathway

This study found that students’ intention to perform implant therapy after graduation was more closely associated with treatment preference, perceived importance of implant therapy, and views on implantology teaching as a separate subject than with academic stage or informal information sources. These findings suggest that professional intentions in implant dentistry may be related not only to information exposure but also to how students conceptualise implant therapy in treatment planning and undergraduate learning. As summarised in Fig. 1, the factors identified in the multivariable model clustered around treatment preference, perceived importance of implant therapy, views on implantology as a separate subject, sex, and implant system familiarity. An extended comparison of the present findings with those of previous studies on undergraduate implant education and student intentions toward implant practice is provided in [Supplementary File 3].

### Treatment preference as a key correlate of future intention

Treatment preference showed the clearest association with future intentions in this study. Students who preferred extraction followed by implant placement were more likely to intend to perform implant therapy after graduation than those who preferred root canal treatment with buildup. This finding suggests that intention may be related not only to knowledge acquisition but also to how students approach treatment planning for compromised teeth. Students who view implants as a preferred restorative option may be more likely to incorporate implant therapy into their future practice. This interpretation is consistent with earlier evidence that structured undergraduate exposure may be associated with subsequent professional engagement in implant dentistry. However, given the cross-sectional design, the observed association should be interpreted as correlational rather than causal. This finding should not be interpreted as support for extraction over tooth preservation. This may indicate that students who perceive implant therapy as a modern and predictable treatment option are more likely to include it in their future practice. In the Iraqi clinical context, where implant treatment is increasingly visible in private practice and patient demand may shape perceptions of contemporary dentistry, students may associate implant therapy with advanced, restorative care. However, this preference may also reflect uncertainty regarding prognosis assessment and the balance between tooth preservation and replacement. Undergraduate teaching should therefore emphasise evidence-based comparisons between endodontic-restorative options and implant-based rehabilitation, including prognosis, biological cost, patient factors, maintenance needs, and referral thresholds.

### Views on implantology as a separate subject

Students who did not support implantology as a separate subject were less likely to report an intention to perform implant therapy after graduation. This finding suggests that students’ views on how implantology should be positioned within undergraduate teaching may be associated with their future professional orientation. One possible interpretation is that students who favour a stand-alone subject may perceive implant dentistry as a more defined and clinically relevant area of practice, whereas others may view it as less central within undergraduate training. This interpretation is broadly consistent with long-standing recommendations to introduce implant dentistry at the undergraduate level as part of comprehensive oral rehabilitation training and with consensus statements that emphasise structured competence in diagnosis, treatment planning, and the restoration of straightforward implant cases in dental education [4–8]. It is also relevant in the context of the persistent variability in implant education across institutions [9, 10]. However, because this was a cross-sectional study, the observed association should not be interpreted causally. Rather, it may reflect broader educational attitudes linked to professional intention.

### Perceived importance and professional intention

Students who rated implants as “very important” or “important” were less likely than those selecting “essential” to report an intention to perform implant therapy after graduation. This pattern was unexpected and should be interpreted cautiously. Possible explanations include differences in how respondents understood the response categories, heterogeneity in the strength of their professional commitment, or residual confounding not captured in the model. Therefore, this finding is better viewed as exploratory rather than as a basis for strong interpretation. One possible explanation is that students who rated implant therapy as “important” or “very important,” but not “essential,” may recognise its clinical value while also perceiving barriers to providing implant therapy after graduation, including surgical complexity, limited confidence, cost considerations, insufficient clinical exposure, or uncertainty regarding the adequacy of undergraduate training. In contrast, students who considered implants “essential” may have had a stronger professional orientation toward implant-related practice. This interpretation remains exploratory and should be examined in future qualitative or longitudinal studies.

### Gender differences

Female students were significantly less likely to report an intention to perform implants (AOR = 0.49; *p* = 0.033). This finding warrants careful interpretation, as previous studies have not consistently shown sex differences in professional intention within implant education [27]. The observed association may reflect differences in confidence, perceived surgical complexity, access to mentorship, procedural opportunities, or sociocultural expectations regarding surgical roles rather than differences in competence. These issues may be particularly sensitive to the local educational context; therefore, they deserve targeted qualitative investigation. Importantly, academic stage was not associated with intention (*p* = 0.542), suggesting that progression through clinical years alone may not substantially alter professional direction in the absence of meaningful curricular exposure.

### Knowledge exposure vs. learning modality

Neither the academic stage nor the primary information source was significantly associated with intention. This suggests that passive or informal exposure through videos, workshops, or social media may be insufficient to shape professional intention on its own. Instead, structured curricular experiences and treatment orientation appear to play a greater role than the learning medium itself. This interpretation is consistent with international studies showing that students often report positive attitudes toward implant dentistry, yet identify deficiencies in practical training, confidence, and preparedness [11–18]. It is also consistent with the broader view that undergraduate dental education is associated with students’ professional outlook and self-reported practice-related intentions beyond technical knowledge alone [28]. In this sense, the present findings may be cautiously considered alongside international discussions on undergraduate implant education [29], because many dental schools face similar challenges in balancing theoretical instruction, practical exposure, and competency development.

### Implant-system familiarity

Implant-system familiarity was associated with the primary multivariable model; however, this relationship was not maintained in the sensitivity analysis, in which familiarity was recoded as any familiarity versus none. This suggests that the original association was not robust and may have been influenced by the category structure, uneven group sizes, or residual confounding. Accordingly, implant-system familiarity should be interpreted with caution and not considered a stable or independent predictor in the present study.

### Educational interpretation and curriculum-related associations

The present findings should be interpreted as curriculum-related associations rather than as evidence of educational effects. The intention to perform implant therapy after graduation was associated with treatment preference, views on implantology teaching as a separate subject, and perceived importance of implant therapy. These associations suggest that students’ professional intentions may be linked to how implant dentistry is positioned within their clinical reasoning and undergraduate learning experiences.

This interpretation is consistent with previous studies reporting associations between predoctoral implant education, perceived preparedness, and students’ future plans to provide implant treatment [27], as well as studies describing graduate practice patterns after exposure to undergraduate implant curricula [30]. However, the cross-sectional design of the present study does not allow us to infer whether curriculum structure caused students’ intentions. Similarly, although supervised implant treatment by undergraduate students has been reported to achieve acceptable clinical outcomes and positive student or patient perceptions [31, 32], the present study did not assess clinical competence or observed practice.

Therefore, the findings should be used mainly to identify areas for further educational evaluation. In practical curriculum terms, these areas may include case-based teaching on implant diagnosis and treatment planning; simulation-based implant planning; comparison of tooth preservation and implant replacement options; restoration-focused implant competencies; recognition of surgical complexity; maintenance and complication awareness; and clear referral thresholds [33]. These elements may help define competence-oriented undergraduate exposure without implying that undergraduate students should independently perform complex surgical implant procedures. Future studies should examine whether structured, competency-based implant education is prospectively associated with changes in students’ confidence, clinical reasoning, treatment planning decisions, and later practice behaviour. The observed sex difference in intention also warrants further qualitative and longitudinal investigation, particularly regarding the development of confidence, access to clinical opportunities, mentorship, and career orientation during dental training. Although the present study was conducted in Iraq, similar questions about undergraduate preparation for contemporary implant-related care may be relevant in other educational settings, provided that local curricular and institutional contexts are considered.

### Limitations

This study had several limitations that should be considered when interpreting the findings. Owing to the cross-sectional design, causal relationships between the factors studied and students’ intentions to perform implant therapy could not be established. The use of electronic distribution channels may have introduced a self-selection bias, as students with a greater interest in implant dentistry may have been more likely to participate. In addition, because the questionnaire was disseminated through student group channels rather than an individual roster, the total number of recipients was unknown, and the response rate could not be determined. Furthermore, because the survey link was accessible through multiple open electronic platforms and was not restricted to a closed institutional sampling frame, an approximate institutional denominator was not reported, as it could have misrepresented the true reach of the survey.

A further important limitation is that 238 of the 542 submitted questionnaires were excluded during screening because they were outside the target eligibility criteria, duplicates, substantially incomplete, lacked interpretable responses for the primary outcome or key covariates, or contained response patterns that could not be reliably interpreted. Although this screening process was intended to improve data quality, the high proportion of excluded submissions may have introduced a selection bias. Therefore, the findings should be interpreted as associations observed in the final eligible complete-case sample rather than as estimates that necessarily represent all Iraqi dental students. No formal a priori sample size calculation was performed; therefore, although the final analytic sample was considered pragmatically adequate for the main regression model, the adequacy of the sample to detect all associations, particularly subgroup comparisons, cannot be assured. The sample was also imbalanced by sex, with substantially fewer male than female participants, which may have reduced the precision of sex-based comparisons. In addition, the questionnaire was self-developed, and several constructs were assessed using single self-report items without formal psychometric validation; therefore, measurement error cannot be ruled out.

The primary outcome was self-reported intention rather than observed future clinical behaviour, which may not fully reflect later professional practice. The multivariable model explained only a limited proportion of the variation in intention, suggesting that other relevant determinants were not captured in the present survey. Finally, the association involving implant system familiarity was not robust to sensitivity analysis and should therefore be interpreted with caution. Because the study was conducted within the context of Iraqi undergraduate dental education, the findings may not be directly generalisable to institutions with different curricular structures or educational environments.

## Conclusions

The null hypothesis was not supported in this study. Future intention to perform dental implant therapy after graduation was associated mainly with treatment preference, perceived importance of implant therapy, and views on implantology as a separate subject, rather than with academic stage or informal learning sources. These findings suggest that students’ views of implant dentistry in undergraduate education are associated with their self-reported professional intentions. Because of the cross-sectional design, these findings should not be interpreted as evidence that curriculum structure determines future practices. Further longitudinal and qualitative studies are needed to examine how undergraduate implant education, confidence, clinical exposure, treatment planning attitudes, and local educational context relate to later implant-related practice.

## Supplementary Information

Supplementary Material 1: STROBE checklist. A PDF table presenting the STROBE criteria and the corresponding manuscript pages where each item is addressed.

Supplementary Material 2: Survey questionnaire. A PDF containing the original questionnaire distributed to participants, together with the indexed keys used for data interpretation.

Supplementary Material 3: Extended comparison of the present findings with previous studies. A PDF presenting an extended comparison of the present findings with previous studies on undergraduate implant education and student intentions toward implant practice.

## Data Availability

The dataset supporting the conclusions of this article is available in the Zenodo repository [34], https://doi.org/10.5281/zenodo.18862096.
