# Supplementary material for: Factors associated with Iraqi dental students’ intention to perform dental implant therapy after graduation: a cross-sectional study
Source: BMC Oral Health. 2026 Jul 21;26:2025. doi: 10.1186/s12903-026-09014-x (PMC13670439; doi:10.1186/s12903-026-09014-x)
Supplement: Supplementary file 1 [file 12903_2026_9014_MOESM1_ESM.pdf]

STROBE Checklist

STROBE item	Requirement	What is currently in the manuscript	Correct page no.
1a	Indicate the study design in the title or abstract	The title states "a cross-sectional study," and the abstract methods also state a cross-sectional analytical study.	1–2
1b	Provide an informative and balanced abstract	The abstract includes objectives, methods, results, and conclusions with the main adjusted findings from the corrected multivariable model.	1–2
2	Explain the scientific background and rationale	The introduction explains the relevance of implant education, the curricular background, and the international and Iraqi contexts.	2–3
3	State-specific objectives and prespecified hypotheses	The aim and null hypothesis are explicitly stated.	3
4	Present key elements of the study design early in the paper	The cross-sectional analytical design is clearly stated at the beginning of the Methods section.	3–4
5	Describe the setting, locations, and relevant dates	The study is described as having been conducted during the 2024–2025 academic year, with the recruitment/data-collection period detailed in the Methods.	4
6a	Give the eligibility criteria and sources/methods of participant selection	Eligible participants were fourth- and fifth-year dental students. Distribution through open electronic student channels and social media platforms is described, and the denominator/non-respondents are stated as unknown.	4–5
6b	Matching criteria, if applicable	Not a matched study.	N/A
7	Clearly define outcomes, exposures, predictors, confounders, and effect modifiers	The primary outcome is clearly defined. Candidate explanatory variables are listed, the final multivariable model variables are specified, and no effect modifiers were prespecified.	4–5
8	For each variable, give data sources and details of measurement	The manuscript describes how the questionnaire was administered and how major variables were measured/categorized. The survey questionnaire is cited in the Methods as Supplementary File S2.	4–5

9	Describe any efforts to address potential sources of bias	Self-selection bias, unknown response rate, and the high exclusion proportion are acknowledged. Screening by three researchers is described, including checks for duplicate submissions, incomplete questionnaires, ineligible responses, and response patterns that could not be interpreted reliably.	5
10	Explain how the study size was arrived at	The manuscript states that no formal a priori sample-size calculation was performed. It reports that 542 submissions were received and that 304 eligible complete responses were retained. A pragmatic sample-size adequacy statement was added, noting that the final sample comprised 304 students, of whom 200 had the outcome event, and that the multivariable model was estimated on 288 students after exclusion of sparse categories. The absence of a formal a priori calculation is acknowledged as a limitation.	4–6, 15
11	Explain how quantitative variables were handled in the analyses	The manuscript states that all analyzed variables were categorical, explains handling of sparse cells, defines reference-category coding, and specifies exclusions from the multivariable model due to very small frequencies.	5–6
12a	Describe all statistical methods, including those used to control for confounding	Chi-square tests, continuity-corrected chi-square for 2 × 2 comparisons, Cramér's V, logistic regression, AORs, 95% CIs, and significance threshold are reported.	5–6
12b	Describe any methods used to examine subgroups and interactions	The manuscript states that no subgroup analyses or interaction analyses were performed and that the additional analysis was a sensitivity analysis.	6
12c	Explain how the missing data were addressed	The manuscript states that incomplete questionnaires were excluded during screening and that only complete eligible responses were included in the final analysis.	5
12d	If applicable, describe analytical methods taking into account the sampling strategy	The manuscript states that recruitment used open electronic channels, no weighting or complex sampling adjustments were applied, and analyses were conducted on an unweighted convenience sample.	5–6
12e	Describe any sensitivity analyses	The sensitivity analysis re-coding implant-system familiarity as "any familiarity" versus "none" is described in the Methods and reported in the Results section.	6, 9
13a	Report the number of individuals at each stage of the study	The manuscript reports the total number of submissions received, screening results, and the final included sample.	4, 6–9

13b	Give reasons for non-participation at each stage	The number and reasons for non-participation could not be determined because the survey was distributed through open electronic student channels and social media platforms rather than a closed, individualized invitation list. The eligible source population could not be reliably defined, and an approximate institutional denominator was not reported because students from other dental colleges may also have accessed the questionnaire. The manuscript separately reports the exclusion criteria for submitted questionnaires, including ineligibility, duplicate submissions, substantial incompleteness, lack of interpretable responses for the primary outcome or key covariates, and unreliable response patterns.	4–6
13c	Consider the use of a flow diagram	A flow diagram was not included. Participant flow is described narratively in the Methods and Results sections, including 542 submissions received, 238 excluded after screening, and 304 retained for the final complete-case analysis.	4–8
14a	Give characteristics of study participants	Table 1 describes demographics and key educational/perceptual variables, and the Results text notes that the sample was predominantly female and fifth-year.	6–8
14b	Indicate the number of participants with missing data for each variable	The manuscript states that incomplete questionnaires were excluded during screening and that only complete eligible responses were included in the final analysis. Variable-specific missing-data counts were not tabulated because excluded questionnaires were not retained as separate analytic categories by variable-specific missingness.	5–6, 15
15	Report the number of outcome events or summary measures	Outcome frequency is clearly reported: 200/304 intended to perform implant therapy after graduation.	6
16a	Give unadjusted estimates and, if applicable, confounder-adjusted estimates with precision	Bivariate associations and adjusted logistic regression with AORs and 95% CIs are presented.	8–9
16b	Report category boundaries when continuous variables were categorized	Variables were collected categorically rather than categorized from continuous scales.	N/A
16c	If relevant, consider translating estimates into absolute risk	Logistic regression of a binary intention outcome; no absolute risk translation is needed.	N/A

17	Report other analyses done, e.g., subgroups and sensitivity analyses	The sensitivity analysis is reported in the Results section.	9
18	Summarize key results with reference to study objectives	The Discussion aligns the findings with the study aim and interprets the main predictors.	10–15
19	Discuss the limitations of the study	A dedicated limitations section is present, covering cross-sectional design, self-selection bias, unknown response rate, exclusion-related selection bias, lack of formal sample-size calculation, sex imbalance, questionnaire validation limitations, self-reported intention, limited explanatory power of the model, instability of implant-system familiarity, and limited generalizability.	15
20	Give a cautious overall interpretation of results	The interpretation is cautious and framed in terms of cross-sectional associations rather than causal effects. The Discussion and Conclusion state that curriculum structure, treatment preference, and perceptual factors were associated with intention, but should not be interpreted as evidence that education causes future practice. Findings related to perceived importance and implant-system familiarity are specifically interpreted with caution.	10–16
21	Discuss generalizability	The manuscript discusses limited generalizability because the study was conducted in the Iraqi undergraduate dental education context using open electronic convenience recruitment. The limitations also include an unknown response rate, a high proportion of exclusions, sex and academic-stage imbalances, and reliance on self-reported intention rather than observed future practice.	14–16
22	Give the source of funding and the role of funders	The Funding section states that the study was not supported by any specific project funding or grant.	17
