# Supplementary material for: Factors associated with Iraqi dental students’ intention to perform dental implant therapy after graduation: a cross-sectional study
Source: BMC Oral Health. 2026 Jul 21;26:2025. doi: 10.1186/s12903-026-09014-x (PMC13670439; doi:10.1186/s12903-026-09014-x)
Supplement: Supplementary file 2 [file 12903_2026_9014_MOESM2_ESM.docx]

| **Serial No.** | **Gender** | **Stage** | **5. In which stage do you study dental implant topic?** | **6. Which stage do you prefer to start studying dental implant dentistry?** | **7. What is the most important source of your information about dental implants?** | **8. What do you think about the importance of dental implant as a treatment?** | **9. Which option do you think is better as a treatment plan for badly destructed tooth?** | **10. Do you think that "Dental Implantology" should be studied as a separate subject in dentistry colleges?** | **11. Do you think it is better to study dental implants with other subjects?** | **12. If your answer is yes, which subject is most important?** | **13. How many dental implant systems do you know?** | **14. Do you plan to perform dental implant after graduation?** |
| --- | --- | --- | --- | --- | --- | --- | --- | --- | --- | --- | --- | --- |

**Indexed answer Keys**

| INDEX: | |
| --- | --- |
| Male | 1 |
| Female | 2 |
| Fourth and Fifth stages | 3 |
| Fourth stage | 4 |
| Fifth stage | 5 |
| Videos | 6 |
| Books and articles | 7 |
| Social media | 8 |
| Workshops | 9 |
| None of the above | 10 |
| Essential | 11 |
| Very Important | 12 |
| Important | 13 |
| Not Important | 14 |
| Not Useful | 15 |
| Root canal treatment and build up (tooth preservation) | 16 |
| Extraction then implant | 17 |
| Extraction then Making a Bridge | 18 |
| Extraction then Making a Denture | 19 |
| Yes | 20 |
| No | 21 |
| Periodontics | 22 |
| Prosthodontics | 23 |
| Crown and bridge | 24 |
| Oral Surgery | 25 |
| One | 26 |
| More than one | 27 |
| No one | 28 |
