# Supplementary material for: Factors associated with Iraqi dental students’ intention to perform dental implant therapy after graduation: a cross-sectional study
Source: BMC Oral Health. 2026 Jul 21;26:2025. doi: 10.1186/s12903-026-09014-x (PMC13670439; doi:10.1186/s12903-026-09014-x)
Supplement: Supplementary file 3 [file 12903_2026_9014_MOESM3_ESM.docx]

**An extended comparison of the present findings with previous studies on undergraduate implant education and student intentions toward implant practice.**

| **Key citation** | **Country/Region** | **Study design** | **Population** | **Focus** | **Key findings** | **Alignment with the present study** |
| --- | --- | --- | --- | --- | --- | --- |
| **American Association of Dental Schools 1991** | USA | Curriculum guideline | Dental schools | Predoctoral implant dentistry recommendations | Early formal guidance supporting implant content in predoctoral curricula | Supports longstanding educational mandate |
| **Lang and De Bruyn 2009** | Europe | Consensus statement | Academic experts | Rationale for curriculum inclusion | Justifies incorporating implant dentistry into dental curricula | Supports need for structured curriculum integration |
| **Sanz and Saphira 2009** | Europe | Consensus statement | Academic experts | Competencies and educational methods | Defines competency expectations and appropriate teaching approaches | Supports competency-based framing |
| **Mattheos et al. 2009** | Europe | Consensus statement | Academic experts | Teaching and assessment | Emphasizes structured teaching and assessment principles | Supports assessment/competency paradigm |
| **Donos, Mardas, and Buser 2009** | Europe | Consensus statement | Academic experts | Postgraduate pathways | Distinguishes undergraduate vs postgraduate pathways and scope | Supports educational ladder and scope boundaries |
| **De Bruyn et al. 2009** | Europe | Cross-sectional survey | Dental schools | Undergraduate implant education provision | Reports substantial heterogeneity in implant teaching across Europe | Confirms global variability in implant education |
| **Koole et al. 2014** | Europe | Follow-up survey | Dental schools | Post-consensus implementation | Continued variability and incomplete harmonization after consensus | Supports ongoing inconsistency |
| **Schweyen et al. 2020** | Germany | Cross-sectional survey | Students, educators, dentists | Attitudes toward implant curriculum | Strong support for implant education; students favor expanded practical exposure | Supports student demand for structured training |
| **Onoriobe et al. 2025** | Nigeria | Mixed methods / cross-sectional surveys | Faculty and students | Curriculum standardization barriers | Resource/faculty/time constraints; strong student desire for implant training | Comparable institutional variability and needs |
| **Yuan et al. 2011** | USA | Cross-sectional survey | Predoctoral dental students | Education and future plans | Perceived preparedness/exposure linked to plans to provide implant treatment | Supports exposure–intention relationship |
| **Mohammed et al. 2025** | Yemen | Multicenter cross-sectional survey | Undergraduate dental students | Educational/clinical training gaps | High awareness, limited practical training; strong demand for training | Matches knowledge–practice gap theme |
| **Moeini et al. 2023** | Iran | Cross-sectional survey | Senior dental students | Knowledge and attitudes | Strong theoretical orientation; limited practical confidence/competence | Supports practical training gap |
| **Saad and Salem 2021** | Saudi Arabia | Cross-sectional web-based survey | Students, interns, recent graduates | Implant complications knowledge/awareness | Variable knowledge of complications; highlights need for curriculum strengthening | Supports structured training/complication preparedness |
| **Sharma et al. 2019** | Nepal | Cross-sectional survey | Undergraduate dental students | Knowledge and perceptions | Knowledge gaps persist; seniority not necessarily linked to adequate knowledge | Supports year-level nonlinearity |
| **Hein et al. 2022** | Myanmar | Cross-sectional survey | Undergraduate dental students | Knowledge and perceptions | Many feel poorly informed; perceived difficulty high; education needed | Reflects confidence barrier and training needs |
| **AlHemrani et al. 2022** | UAE | Cross-sectional survey | Students and interns | Knowledge and perceptions | Knowledge improves with training level but gaps remain | Reinforces exposure-quality effect |
| **Huebner 2002** | USA | Graduate follow-up / retrospective comparison | Dental graduates | Impact of predoctoral implant curriculum on practice | Clinical/lab exposure associated with higher implant-related practice engagement after graduation | Supports training → later practice pattern link |
| **Vandeweghe et al. 2014** | Belgium | Prospective clinical study | Undergraduates + patients | Student-placed implants outcomes | Acceptable outcomes and patient/student satisfaction under supervision | Supports feasibility of supervised clinical exposure |
| **Kämmerer et al. 2021** | Germany | Prospective clinical implementation | Students + patients | Supervised mini-implant overdenture program | High survival and positive educational impact | Supports safe supervised training model |
| **Goey et al. 2025** | Netherlands | Cross-sectional survey | Students & dental professionals | AI acceptability | Positive acceptability; supports role of AI tools in dentistry | Context for technology-enhanced education |
| **Ghasemian et al. 2025** | Iran | Cross-sectional survey | Dental students | AI attitudes/perceptions | High perceived impact; supports need for AI/digital literacy discussion | Supports modern learning environment context |
| **Fischer et al. 2024** | Germany | Educational intervention evaluation | Dental students | Structured implant training program | High acceptance; desire for more practical training | Supports structured curricular innovation |
| **Ziada et al. 2024** | USA | Qualitative study | Dental students | Reflection on preclinical implant education | Reflective learning supports confidence/understanding | Supports experiential learning argument |
| **Jyoti et al. 2025** | India | Cross-sectional survey | Practicing dentists | Immediate implant placement attitudes/knowledge | Highlights knowledge–confidence gaps | Supports need for systematic foundational education |
| **Veerabhadrappa et al. 2025** | Malaysia | Cross-sectional survey | Clinical-year dental students | AI perceptions (general dentistry context) | Positive attitudes toward AI; educational integration interest | Supports broader digital/AI educational context |
| **Cheng et al. 2026** | Malaysia | Cross-sectional survey | Graduating dental students | Implant education sufficiency | Perceived insufficient practical exposure; desire for enhanced training | Matches perception gap |
| **Dragan et al. 2019** | International / Europe-centered professional sample | Cross-sectional survey | Practicing dentists | First implant experience and educational pathway | Dentists’ early implant experiences and later implant-related trajectories were influenced by educational background, time in practice, and training pathway; the authors emphasized the need for quality-assured implant education | Supports the broader link between educational pathway and later implant-related professional development |
| **Chaturvedi et al. 2021** | India | Cross-sectional educational evaluation/survey | Dental students/graduates | Perceptions and attitudes toward predoctoral implant education techniques | Structured and modified implant teaching approaches improved interest, knowledge, and attitudes; earlier and broader integration of implant topics was recommended | Supports structured and interactive teaching approaches and earlier curricular integration |
| **Present study** | Iraq | Cross-sectional | Clinical-year dental students | Intention and determinants | Treatment philosophy and curricular integration predict intention | Extends evidence to Iraqi context |
